# Supplementary figures and images for: Evaluation of the accuracy of imputed sequence variant genotypes and their utility for causal variant detection in cattle
Source: Genet Sel Evol. 2017 Feb 21;49:24. doi: 10.1186/s12711-017-0301-x (PMC5320806; doi:10.1186/s12711-017-0301-x)

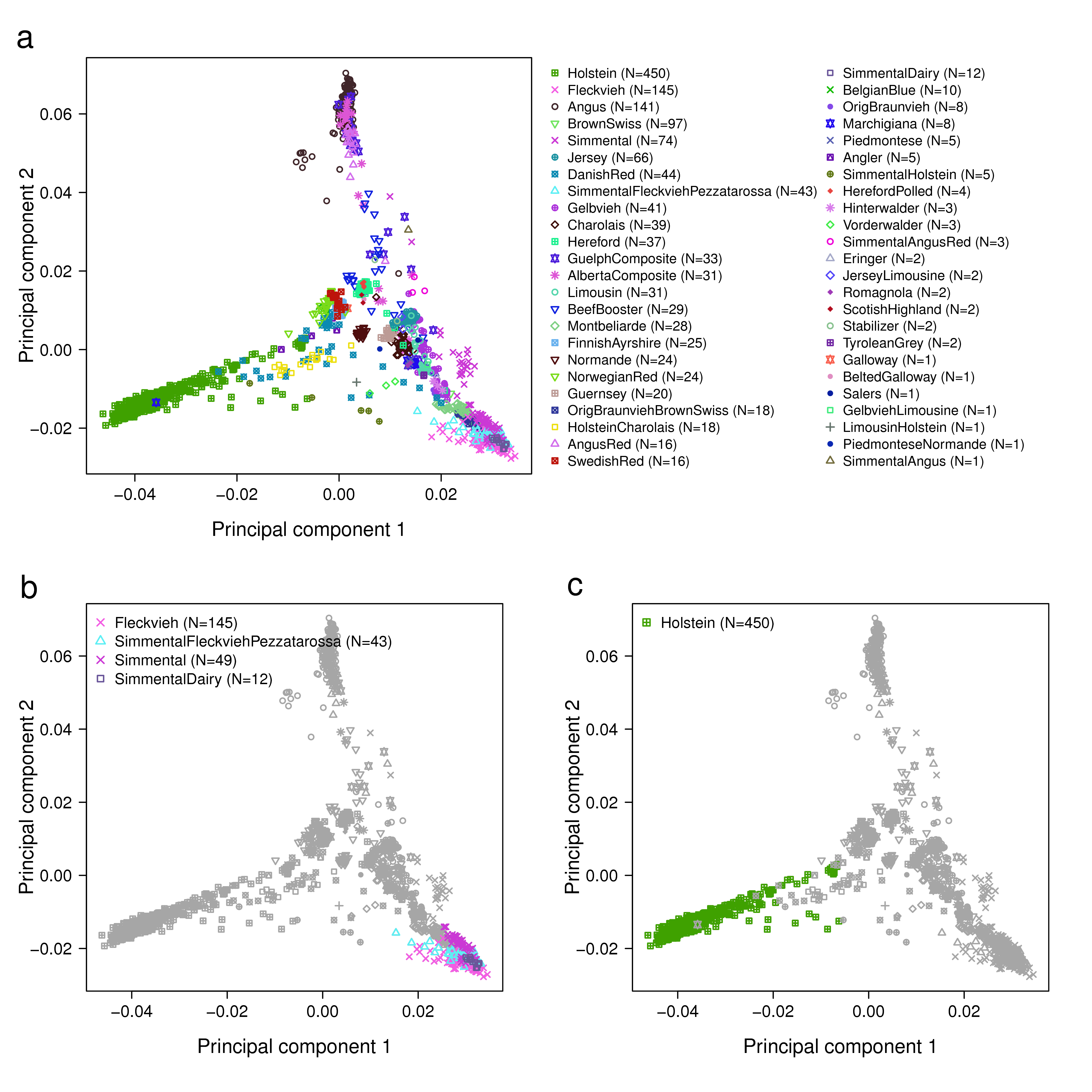

Supplement: Supplementary file 1 — Additional file 1: Figure S1. Principal component analysis in 1577 sequenced animals. a–c Plot of the top two principal components of the genomic relationship matrix. Different colours and symbols represent different breeds. The partners of the 1000 bull genomes consortium assigned the animals to breeds. b Non-grey symbols indicate 249 animals that were considered as Fleckvieh animals. c Green symbols indicate 450 Holstein animals. [file 12711_2017_301_MOESM1_ESM.tif]

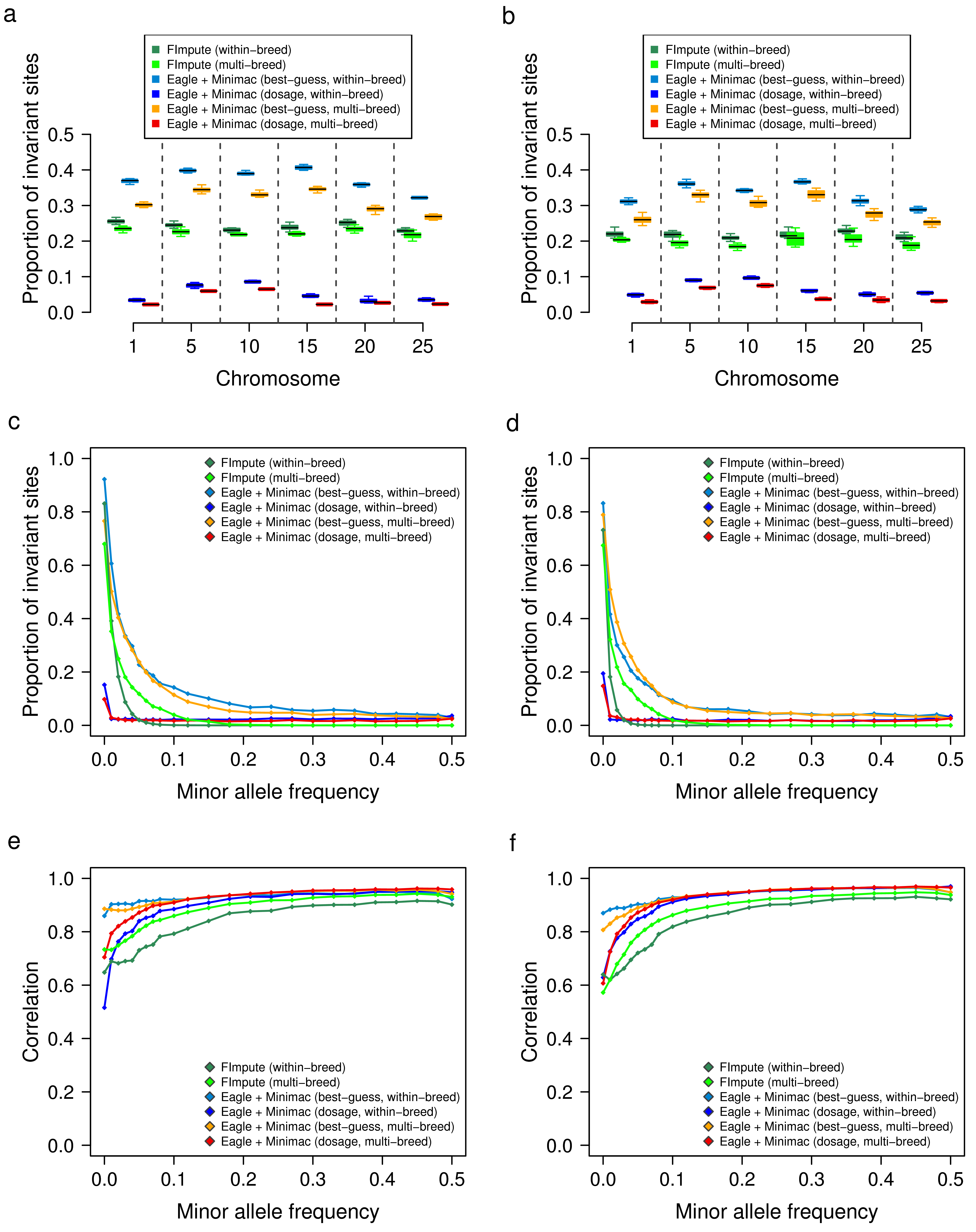

Supplement: Supplementary file 2 — Additional file 2: Figure S2. Sequence variants that were invariant in the validation populations. a–d Proportion of sequence variants that were invariant after imputation in the FV (a, c) and HOL (b, d) validation populations for six chromosomes analysed and different MAF classes. e, f The correlation between imputed and true genotypes was calculated using only sequence variants that were polymorphic in the FV (e) and HOL (f) validation populations. [file 12711_2017_301_MOESM2_ESM.tif]

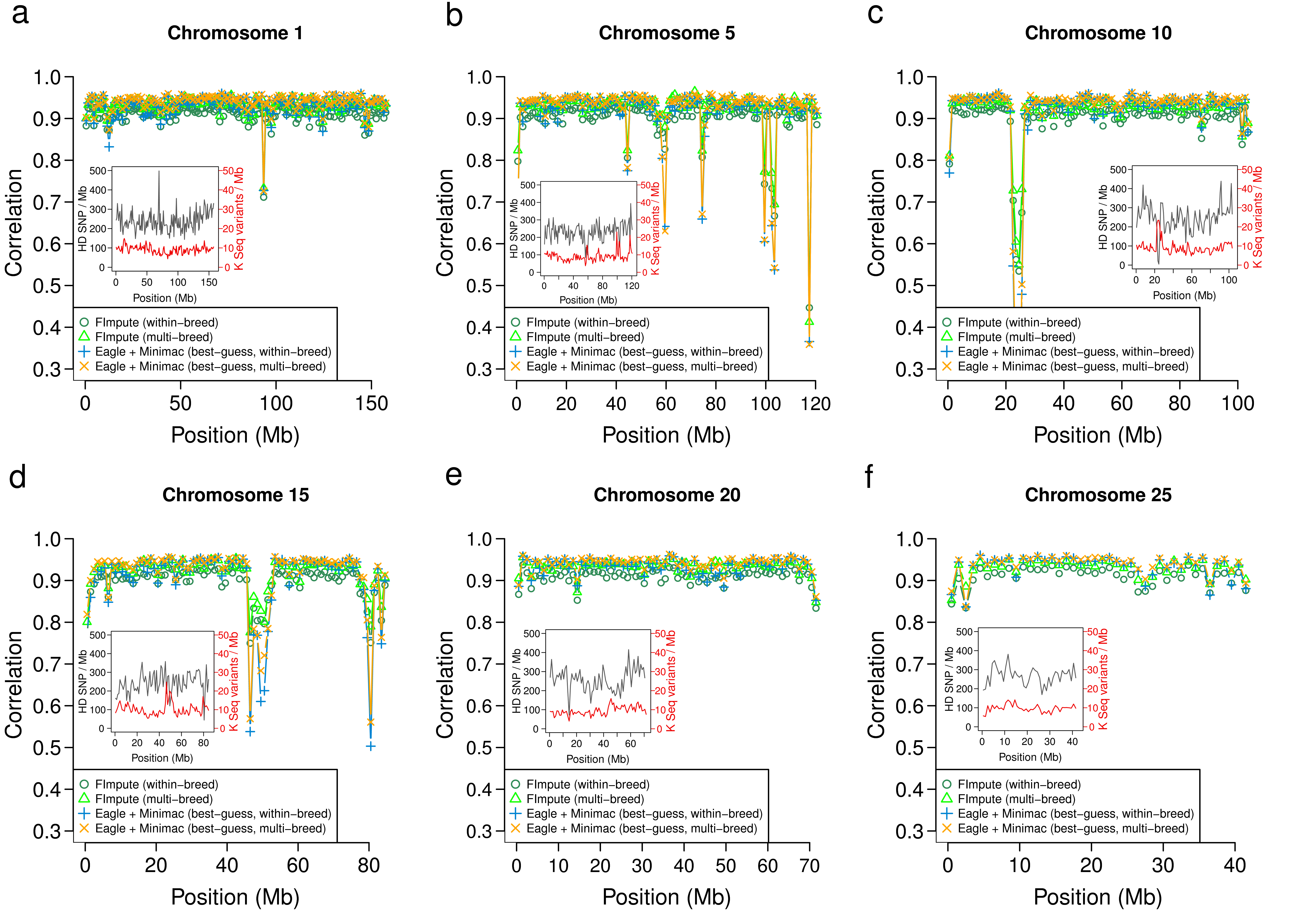

Supplement: Supplementary file 3 — Additional file 3: Figure S3. Imputation accuracy along six chromosomes in Fleckvieh cattle. a–f The correlation between true and imputed genotypes for sequence variants located within successive 1-Mb windows on six chromosomes. Different colours and symbols represent correlation coefficients obtained using different imputation scenarios. Insets HD SNP coverage and sequence variant density along the chromosome. Black and red colours represent the number of SNPs that were included in the BovineHD Bead Chip (HD) and sequence (Seq) variants (×1000, (K)) that were polymorphic in the multi-breed reference population, respectively, per million basepairs (Mb). [file 12711_2017_301_MOESM3_ESM.tif]

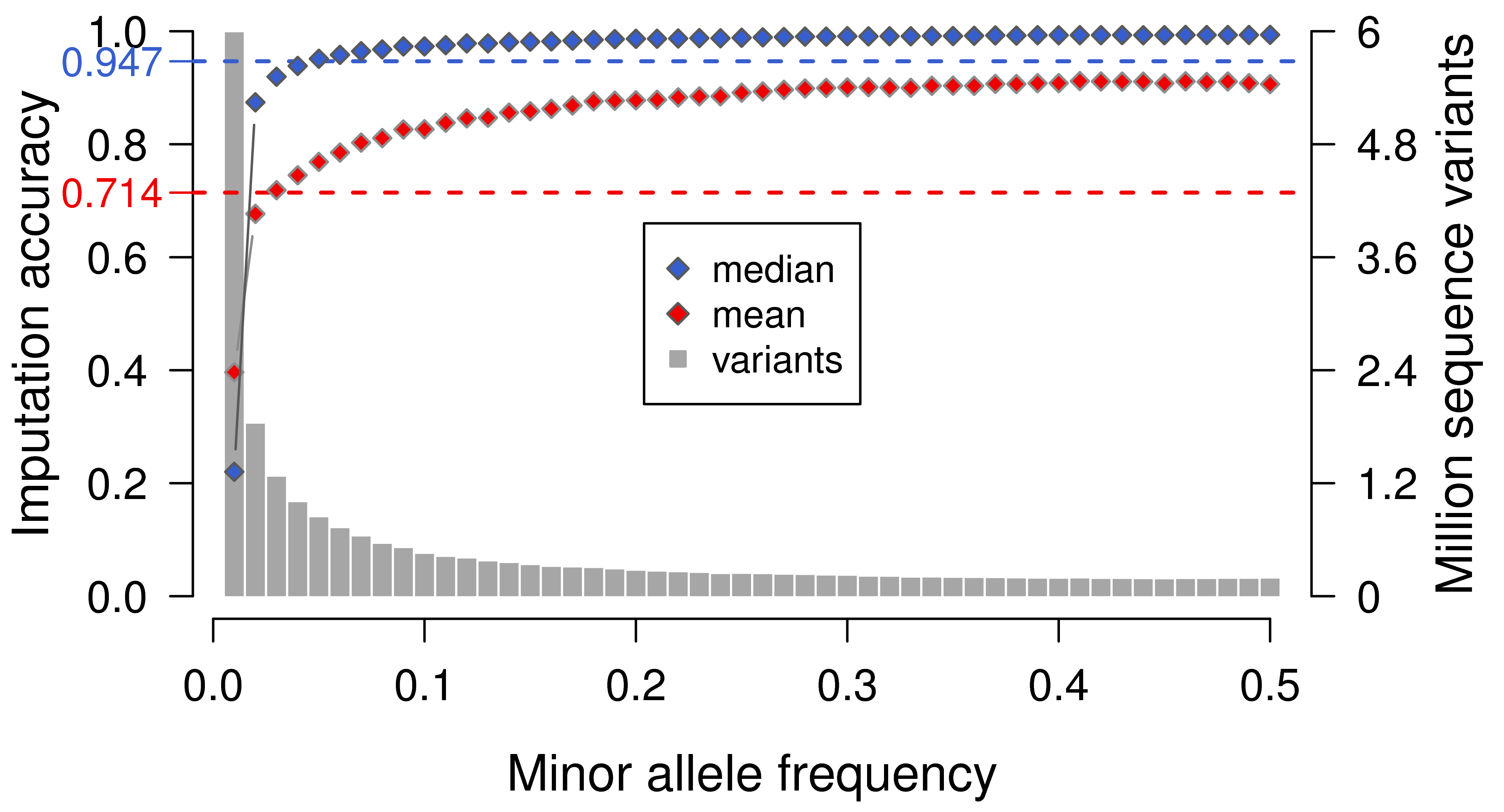

Supplement: Supplementary file 4 — Additional file 4: Figure S4. Accuracy of imputation for 23,256,743 sequence variants in 6958 animals. The mean (red) and median (blue) accuracy of imputation (r2-values were obtained using Minimac) for 23,256,743 sequence variants (grey bars) as a function of the minor allele frequency. The dotted lines represent the mean and median accuracy of imputation across all sequence variants. [file 12711_2017_301_MOESM4_ESM.tif]

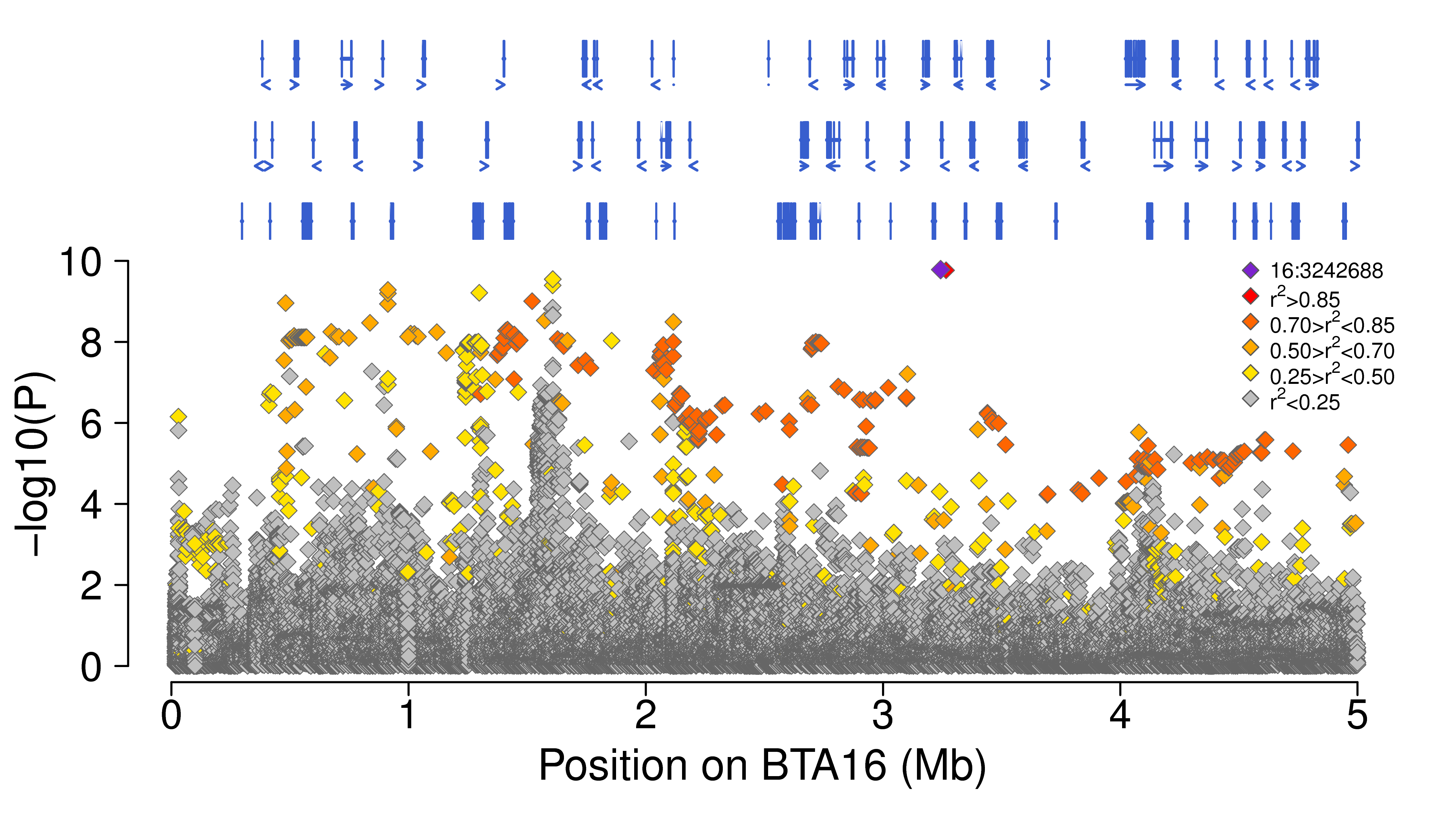

Supplement: Supplementary file 6 — Additional file 6: Figure S5. Detailed view of a milk fat percentage QTL on bovine chromosome 16. Different colours represent the linkage disequilibrium between the most significantly associated variant (violet) and all other variants. Blue arrows indicate the direction of the gene transcription. [file 12711_2017_301_MOESM6_ESM.tif]

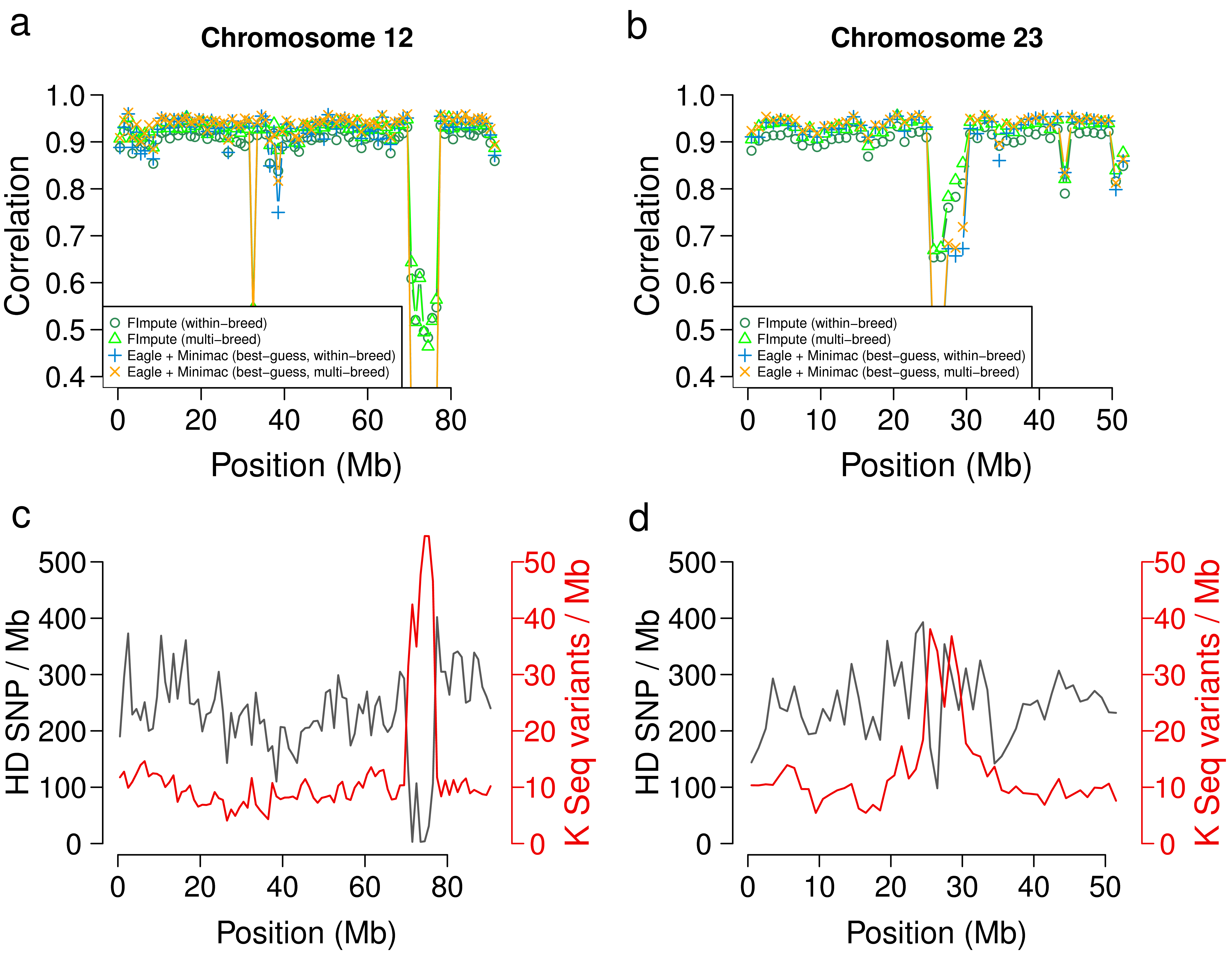

Supplement: Supplementary file 7 — Additional file 7: Figure S6. Imputation accuracy along chromosomes 12 and 23 in Fleckvieh cattle. a, b Correlation between true and imputed genotypes for sequence variants located within successive 1-Mb windows on chromosomes 12 and 23. Different colours and symbols represent correlation coefficients obtained using different imputation scenarios. c, d Red colours represent the number of SNPs that were included in the BovineHD Bead Chip (HD) and sequence (Seq) variants (×1000 (K)) that were polymorphic in the multi-breed reference population, respectively, per million basepairs (Mb). We were eventually able to impute sequence variants for BTA12 and 23 using FImpute when we discarded sequence variants that were located between 70 and 77 Mb and between 25 and 30 Mb, respectively, from the reference panel. [file 12711_2017_301_MOESM7_ESM.tif]

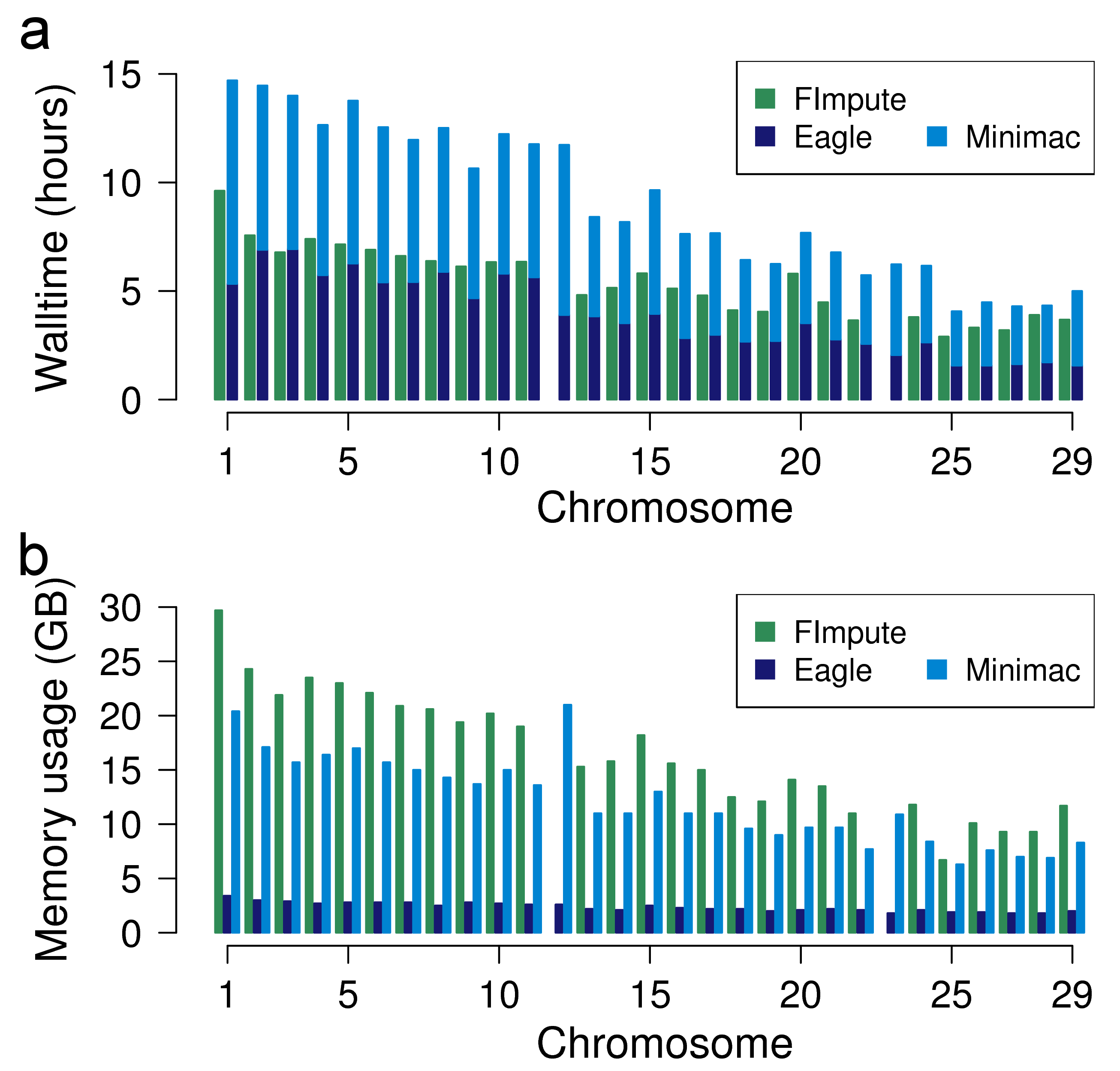

Supplement: Supplementary file 8 — Additional file 8: Figure S7. Computing resources required to impute 23,256,742 sequence variants in 6958 animals. The wall-clock times (a) and random-access memory (RAM) usage (b) required to infer haplotypes and genotypes with FImpute (green), Eagle (dark blue) and Minimac (light blue) were assessed on 12-core Intel® Xeon® processors rated at 2.93 GHz with 96 GB of RAM. FImpute ran out of memory and did not finish when we attempted to infer genotypes for BTA12 and BTA23. FImpute was run on a single processor whereas Eagle and Minimac used 10 processors per chromosome. [file 12711_2017_301_MOESM8_ESM.tif]

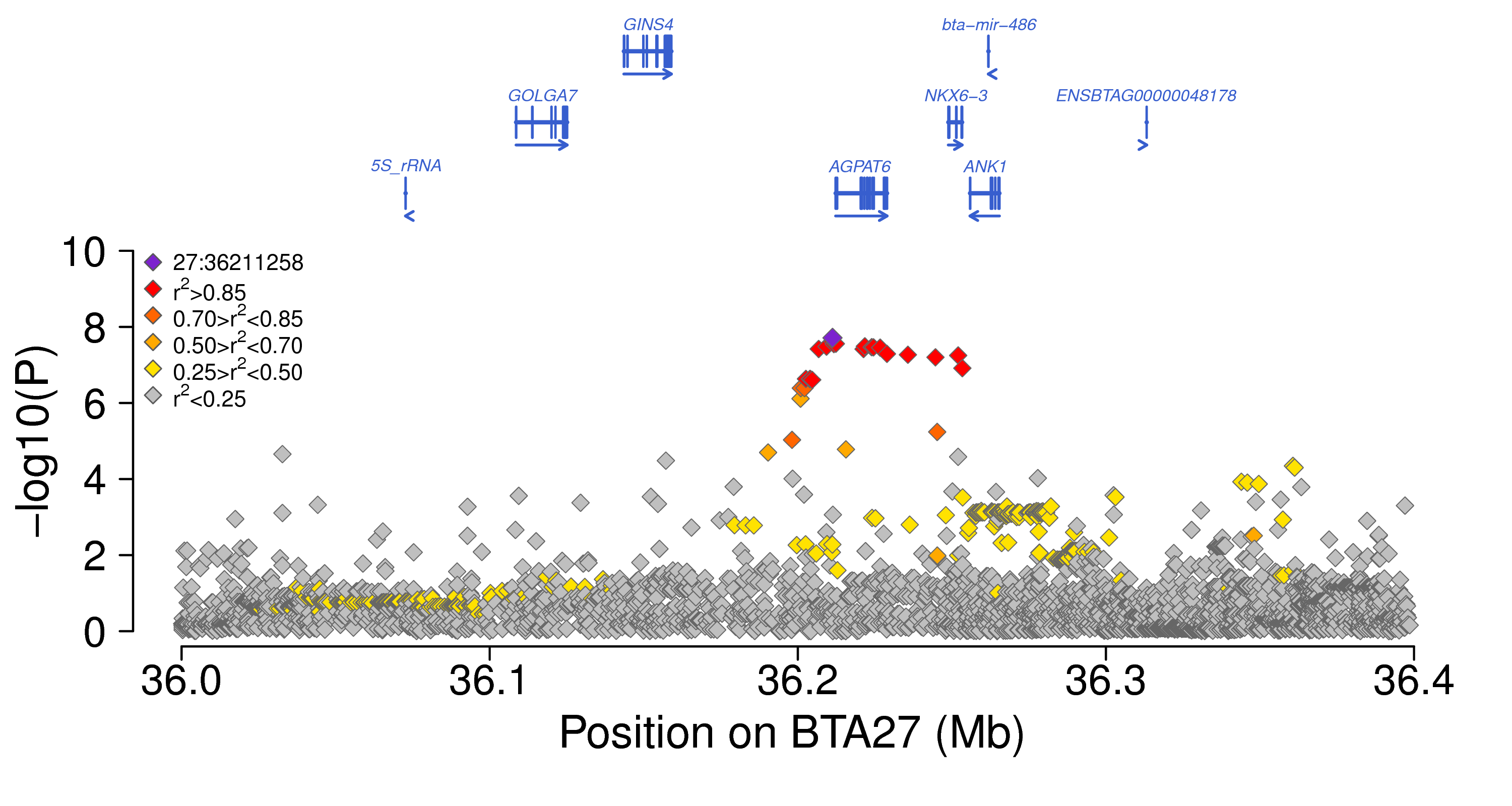

Supplement: Supplementary file 9 — Additional file 9: Figure S8. Detailed view of a milk fat percentage QTL on bovine chromosome 27. Different colours represent the linkage disequilibrium between the top variant (violet) and all other variants. Blue arrows indicate the direction of the gene transcription. The top variant (36,211,258 bp) was associated with fat percentage (P = 1.9 × 10^−8^) albeit not at the genome-scale. Twenty-two variants in high LD (r2 > 0.68) with the top variant were located between 36,200,888 and 36,253,406 bp and had P values less than 7.7 × 10^−7^. Among those were three candidate causal variants (36,211,252 bp with P = 2.4 × 10^−8^, 36,211,708 bp with P = 2.8 × 10^−8^, 36,209,319 bp with P = 3.3 × 10^−8^) for fat content in the early lactation that were reported in Daetwyler et al. [2]. [file 12711_2017_301_MOESM9_ESM.tif]
